# Effect of Sn Content on Wettability and Interfacial Structure of Cu–Sn–Cr/Graphite Systems: Experimental and First-Principles Investigations

**DOI:** 10.3390/ma18081793

**Published:** 2025-04-14

**Authors:** Wenjuan Ci, Qiaoli Lin, Xuefeng Lu, Yu Shi, Likai Yang, Wenkai Wang

**Affiliations:** State Key Laboratory of Advanced Processing and Recycling of Nonferrous Metals, Lanzhou University of Technology, No. 287 Langongping Road, Lanzhou 730050, China; ciwenjuan@outlook.com (W.C.); lqllinqiaoli@163.com (Q.L.); lxfeng@lut.edu.cn (X.L.); yanglikai@lut.edu.cn (L.Y.); 201080503003@lut.edu.cn (W.W.)

**Keywords:** wettability, surface energy, interface energy, adhesion work, stability, DFT

## Abstract

The co-addition of chromium (Cr) and tin (Sn) is known to enhance the wettability between copper (Cu) and graphite (C_gr_), but the effect of Sn content remains poorly understood. This study aims to systematically investigate the influence of Sn content *a* (*a* = 0, 10, 20, 30, 40, 50, 80, 99 at. %) on the wettability, interfacial structure, surface/interface energy (*σ*_lv_/*σ*_sl_), and adhesion behavior of the Cu–*a*Sn–1Cr/C_gr_ system at 1100 °C. The experimental results show that as the Sn content increases, the equilibrium contact angle (*θ*_e_) of the metal droplet shows a non-monotonic trend; the thickness of the reaction product layer (RPL, consisting of Cr carbides (Cr*_m_*C*_n_*)) gradually increases, accompanied by a decrease in the calculated adhesion work ($W_{\mathrm{ad}}^{\mathrm{cal}}$). A “sandwich” interface structure is observed, consisting of two interfaces: metal||Cr*_m_*C*_n_* and Cr*_m_*C*_n_*||C_gr_. Sn content mainly affects the former. At metal||Cr*_m_*C*_n_*, Sn exists in various forms (e.g., Cu–Sn solid solution, Cu*_x_*Sn*_y_* compounds) in contact with Cr*_m_*C*_n_*. To elucidate the wetting and bonding mechanisms of metal||Cr*_m_*C*_n_*, simplified interfacial models are constructed and analyzed based on first-principles calculations of density functional theory (DFT). The trend of theoretically calculated results (*σ*_metal_ and *W*_ad_) agrees with the experimental results (*σ*_lv_ and $W_{\mathrm{ad}}^{\mathrm{cal}}$). Further analysis of the partial density of state (PDOS) and charge density difference (CDD) reveals that charge distribution and bonding characteristics vary with Sn content, providing the microscopic insight into the nature of wettability and interfacial bonding strength.

## 1. Introduction

Graphite (C_gr_) has low density, high thermal conductivity, low thermal expansion coefficient, high-temperature resistance, and self-lubricating properties [1]. Copper (Cu) is commonly used to prepare composites with C_gr_ due to its economic feasibility, easy availability of resources, and excellent alloy solubility. With the continuous development of Cu/C_gr_ composites, their mechanical and functional properties have attracted increasing attention [2,3,4]. However, the applications of Cu matrices are limited by their inherent low hardness and strength, as well as poor creep and corrosion resistance. Recently, alloying copper matrix to prepare Cu/C composites has become a promising research hotspot. The addition of Sn to the Cu matrix significantly enhances its strength, wear resistance, and corrosion resistance through solid solution strengthening. Moreover, Sn improves the functional properties of both Cu/C_gr_ composites and brazed joints, such as electrical and thermal conductivity. These performance improvements are closely dependent on the Sn content [5,6,7,8,9]. Therefore, optimizing the Sn content is critical for enhancing the performance of Cu–Sn/C_gr_ composites and solder joints. It is expected to demonstrate significant potential in a wide range of advanced applications, including construction machinery, electronic packaging, sliding conductive components, and high-efficiency thermal management systems.

Good wettability and strong interfacial bonding are essential for brazed joints and Cu/C composites. However, the intrinsic wettability of Cu–Sn/C_gr_ is poor [10], and the bonding is weak due to van der Waals forces, making reliable connections difficult. Two approaches are commonly used to improve wettability: surface modification of carbon materials and copper-based alloying. The latter is more practical, as surface modification often struggles with coating uniformity and reliability. Copper-based alloying involves adding carbide-forming elements such as Ti, Cr, V, Mn, Nb, W, Mo, or Zr [11] to enhance interfacial reactivity. Among them, Cr is particularly effective. Most studies focus on Cu–Cr/C systems, with limited attention to the role of Sn. References [12,13] showed that adding a small amount of Cr significantly improves Cu/C interfacial wettability. However, Cr’s high reactivity causes rapid formation of dense carbides (Cr*_m_*C*_n_*), which hinders infiltration. Lin et al. [14] found that adding Sn to Cu–Cr alloys enhances wettability on porous C_gr_ by modifying Cr reactivity and reducing droplet surface energy, resulting in deeper infiltration. Thus, Sn content can effectively regulate wetting behavior. Moreover, Sn facilitates low-temperature joining, expanding the processing window for Cu/C composite preparation via melt infiltration.

Hsieh et al. [15] showed that Sn addition in Cu–Ti alloys lower the melting point and affects alloy microstructure and properties. As alloy complexity increases, interfacial structures become more intricate, affecting the interfacial bonding properties (IBPs) (abbreviations in this paper can be found in Supplementary Materials). Optimizing wettability and IBPs typically requires extensive trial-and-error experiments, which are time-consuming and inefficient. Thermodynamic calculations offer qualitative insights, but the underlying mechanisms remain unclear. Moreover, key parameters related to IBPs are environment-sensitive and difficult to measure directly. First-principles calculations based on density functional theory (DFT) have been widely used to study wettability and bonding behavior without empirical inputs [16]. A commonly used approach is the solid/solid slab model, which approximates the liquid/solid interface by neglecting atomic thermal vibrations at high temperatures [17]. When the trends from theoretical and experimental results are consistent, correction methods can be applied to improve accuracy [18]. Despite its advantages, this method is limited by computational cost, as only small-scale systems (typically 100–200 atoms) can be modeled. DFT studies are typically limited to simple interfaces involving pure metals or compounds and cannot easily handle large supercell models or multicomponent alloys. Meanwhile, extensive research exists on non-reactive metal/carbide interfaces (e.g., Al/TiC [19], Co/WC [20], Ag/Ti (C, N) [21], etc.).

This study investigates the wettability and interfacial behavior of Cu–*a*Sn–1Cr alloys on C_gr_ using a modified sessile drop method, which minimizes pre-reaction and oxidation during heating. The interfacial structure and elemental composition of the samples are characterized using a scanning electron microscope (SEM) equipped with energy-dispersive spectroscopy (EDS). Typical interfacial configurations observed experimentally are then selected for simplified DFT modeling, aiming to uncover the mechanisms by which alloying elements and their concentrations influence wettability and IBPs. The results offer both experimental and theoretical guidance for designing Cu–Sn/C composites and solder alloys, and provide a reference for incorporating other non-reactive, low-melting-point elements (e.g., gallium, germanium, silver, indium, lead) into the Cu matrix.

## 2. Experimental and Calculation

### 2.1. Experimental Materials and Methods

The C_gr_ substrate has a purity of >99.9%, a size of 20 mm × 20 mm × 5 mm, an ash content of <40 ppm, a density of ~1.85 g/cm^3^, a porosity of 13%, and a surface roughness (*R*a) of approximately 30–50 nm. The metallic materials used include Cu and Sn foils with a purity of 99.99% and Cr powder with a particle size of 400 μm. The Cu–*a*Sn–1Cr alloys (*a* = 0, 10, 20, 30, 40, 50, 80, 99 at. %) are prepared based on atomic ratios. High-purity Ar gas (99.999%) is repeatedly introduced into the KDH-300B mini vacuum arc furnace, equipped with Ti getters, to purify the furnace chamber. To ensure the uniformity of alloy melting, the wrapped metal is first melted into spheres in an Ar atmosphere before using a robotic arm to flip the alloy and continue the melting process. This procedure is repeated 3–5 times. After the melting, the sample is cooled to room temperature under flowing Ar at a rate of ~15 °C/min. Based on the modified sessile drop method, the metal is first stored in a stainless-steel tube outside the furnace. The furnace is evacuated to ~6–7 × 10^−^⁴ Pa, and the chamber temperature is then heated to the preset experimental temperature of 1100 °C at a rate of 20 °C/min. After the dynamic vacuum is maintained at a stable level (~3–4 × 10^−4^ Pa), the metal is dropped onto the C_gr_ surface through an open alumina tube. Simultaneously, a high-resolution charge-coupled device (CCD) camera is used to record the dynamic spreading and wetting process of the molten metal, with a holding time of 30 min. The specific experimental setup and procedure are referenced in Ref. [22]. The contact angle (CA) in the photographs is measured using the Surface Meter software (SM, version 1.1.0.1, developed by NB Scientific Instruments Co., Ltd., Ningbo, China), and the variation curve of CA with time is obtained. The modified sessile drop method, compared to the traditional sessile drop method, effectively avoids the pre-reaction between the melt and the substrate during the experimental heating process. To prevent oxidation of the metal droplets, the droplets are first cooled to 600 °C under high vacuum, followed by a cooling process at a rate of 15 °C/min with flowing Ar until room temperature. The interface structure, microstructure, morphology of reaction products, and microregional elemental composition of the samples’ cross-section are analyzed and characterized using a Scanning Electron Microscopy (SEM, FEG 450, Thermo Fisher Scientific, Waltham, MA, USA) equipped with an Energy Dispersive Spectrometer (EDS, Oxford Instruments, Abingdon, UK) with a spot size of approximately 2 μm. The samples for high-resolution transmission electron microscope (HRTEM, JEOL, JEM–F200, Tokyo, Japan) are prepared using a focused ion beam (FIB; Helios G4 PFIB, Thermo Fisher Scientific, Waltham, MA, USA).

### 2.2. Calculation Methods

The calculations in this paper are performed using the Cambridge Sequential Total Energy Package (CASTEP, version 22.11, BIOVIA, San Diego, CA, USA) software, which is based on first-principles density functional theory [23]. The Perdew–Burke–Ernzerhof (PBE) function within the Generalized Gradient Approximation (GGA) is used to describe the exchange-correlation energy of Cu ([Ar] 3*d*^10^4*s*^1^), Sn ([Kr] 4*d*^10^5*s*^2^5*p*^2^), Cr ([Ne] 3*s*^2^3*p*^6^3*d*^5^4*s*^1^), and C ([He] 2*s*^2^2*p*^2^), respectively. The OTFG ultrasoft pseudopotentials are employed to model the interaction between atomic nuclei and valence electrons [24], and the energy cutoff is set as 500 eV. The energy fluctuation threshold for static self-consistent calculations is set to 2 × 10^−5^ eV/atom. The Broyden–Fletcher–Goldfarb–Shanno (BFGS) algorithm is used for structure optimization, and the iterative self-consistent convergence criterion is that the energy fluctuation per atom is less than 1 × 10^−5^ eV. The interatomic force is less than 0.05 eV/Å. The maximum stress is below 0.05 GPa, and the atomic displacement fluctuation is less than 1.0 × 10^−3^ Å [25]. Using the density of 0.04 Å^−1^ Monkhorst-Pack *k*-point for Brillouin zone sampling for the bulk, surface, and interface. The bulk models are presented in Figure S1 in Supplementary Materials. In the interface calculations, the interactions among surface layer atoms play a crucial role, while fixing the topmost and bottom layers of atoms ensures the convergence of energy.

## 3. Results

### 3.1. Wetting Experiment

**Figure 1:**
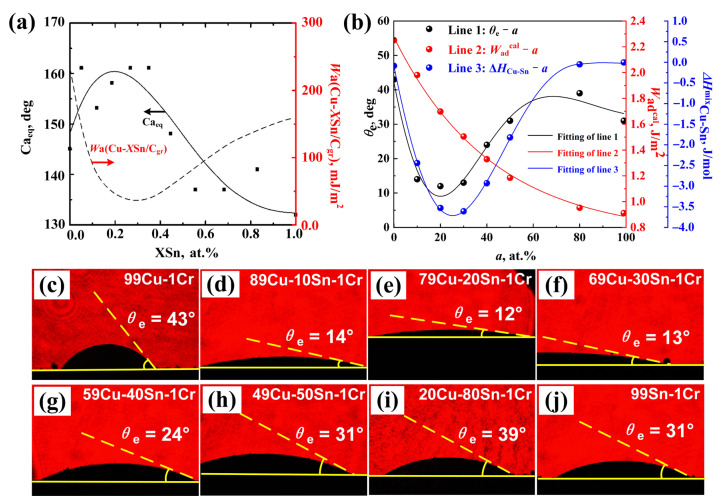
Variation of wetting parameters: (**a**) the variation of CA_eq_ and *W*_ad(Cu–*X*Sn/Cv)_ with Sn content (*X* at. %) of Cu–Sn alloy on the C_V_ at 1150 °C [26]; (**b**) variation of *θ*_e_, $W_{\mathrm{ad}}^{\mathrm{cal}}$, and *ΔH^mix^*_Cu–Sn_ [32] with Sn content; (**c**–**j**) measured results of *θ*_e_ with Sn content.

Eustathopoulos et al. [26] have systematically analyzed the equilibrium contact angle (CA_eq_) and adhesion work (*W*_a(Cu–*X*Sn/Cv)_) of Cu–*X*Sn alloys on the vitreous carbon (Cv) surface as a function of Sn content, as shown in Figure 1a. The results show that CA_eq_ increases first and then decreases with increasing Sn, while *W*_a(Cu–*X*Sn/CV)_ exhibits an inverse trend. In this study, after adding 1 at. % Cr, the measured *θ*_e_ (Figure 1b) follows a different trend—decreasing first, then increasing, and finally decreasing again. When Sn reaches 20 at. %, *θ*_e_ drops to 12°. According to Dupre’s definition of the adhesion work $W_{\mathrm{ad}}^{\mathrm{cal}}$ at solid/liquid interfaces, as shown in Equation (1) [27]:

(1) $$W_{\mathrm{ad}}^{\mathrm{cal}}\;=\sigma_{\mathrm{lv}}\;+\;\sigma_{\mathrm{sv}}\;-\;\sigma_{\mathrm{sl}}$$

where *σ*_lv_ represents the surface energy of the liquid, *σ*_sv_ represents the surface energy of the solid, and *σ*_sl_ represents the solid/liquid interface energy. However, the value of $W_{\mathrm{ad}}^{\mathrm{cal}}$, *σ*_sv_, and *σ*_sl_ cannot yet be accurately measured through experiments. Therefore, we used experimentally measured wetting parameters to estimate the adhesion work $W_{\mathrm{ad}}^{\mathrm{cal}}$ based on the well-known Young–Dupre Equation (2) [28]:

(2) $$W_{\mathrm{ad}}^{\mathrm{cal}}\;=\sigma_{\mathrm{lv}}\;(1\;+\;\cos\;\theta_{e})$$

where *σ*_lv_ represents the surface energy of the Cu–Sn metallic melt, and its values under different Sn contents are listed in Table 1, based on data reported in Ref. [29]. *θ*_e_ is expressed in radians. The measured values of *θ*_e_ are shown in Figure 1c–j. Substituting *σ*_lv_ and *θ*_e_ into Equation (1) yields the $W_{\mathrm{ad}}^{\mathrm{cal}}$, and the variation of $W_{\mathrm{ad}}^{\mathrm{cal}}$ with Sn content is shown as the blue curve in Figure 1b. Compared with the Cu–*X*Sn/Cv system, *θ*_e_ is generally smaller and *W*_a_ (0.8–2.4 J/mol) is significantly larger than that of the Cr-free case (0.1–0.3 J/mol). This suggests that Cr addition enhances wettability and interfacial bonding. Lin et al. [14] attributed this wetting behavior to the decrease surface energy (*σ*_lv_) of the alloy melt with increasing Sn content and a shift in reaction products from Cr_7_C_3_ (major) + Cr_3_C_2_ (minor) to Cr_7_C_3_ (minor) + Cr_3_C_2_ (major). This indicates that Sn affects both melt properties and solid/liquid interface energy (*σ*_sl_). Unfortunately, they did not provide an in-depth explanation for this. Beyond *σ*_lv_, Sn content also influences the alloy’s density, viscosity, and mixing enthalpy (*ΔH*^mix^_Cu–Sn_). According to References [30,31], both the density and viscosity of the Cu–Sn melt decrease with increasing Sn content, but do not explain the trend in *θ*_e_. Interestingly, the variation of *ΔH*^mix^_Cu–Sn_ [32] aligns well with *θ*_e_ (blue curve, Figure 1b). There may be a relationship between *ΔH*^mix^_Cu–Sn_ and *θ*_e_, as *ΔH*^mix^_Cu–Sn_ influences the *σ*_lv_ and *σ*_sl_ of the melt droplet. However, no relevant studies to date have demonstrated a relationship between the two. This may be related to the fact that the complex mechanisms of action within the alloy and at the interface between the alloy and the reaction products have not yet been solved. Therefore, this study will attempt to explain the relationship between alloying elements and their content variations with wettability and IBPs, and their potential mechanisms in Section 4.

### 3.2. Interfacial Structure

**Figure 2:**
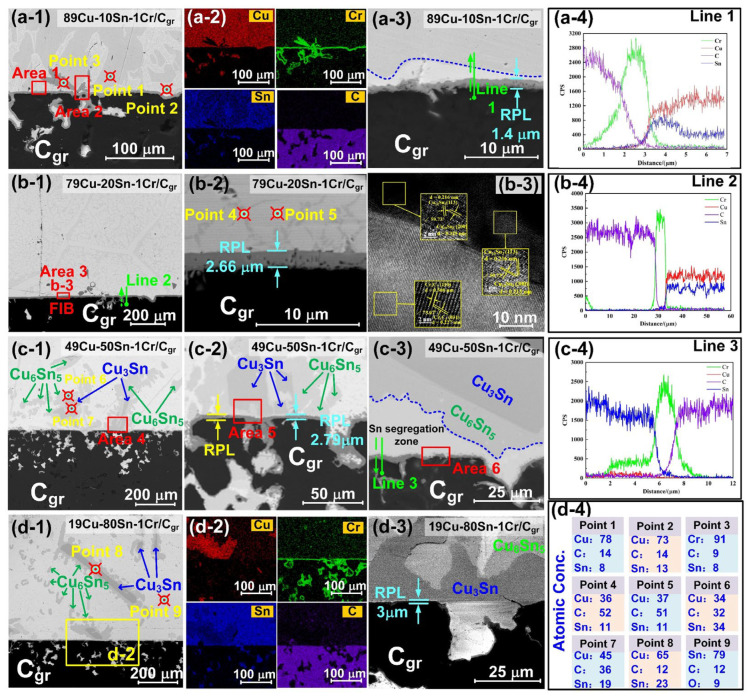
Microstructure near the cross-sectional interface of Cu–*a*Sn–1Cr/C_gr_ (backscattered mode): (**a-1**–**a-4**) 89Cu–10Sn–1Cr/C_gr_, (**b-1**–**b-4**) 79Cu–20Sn–1Cr/C_gr_, (**c-1**–**c-4**) 49Cu–50Sn–1Cr/C_gr_, (**d-1**–**d-4**) 19Cu–80Sn–1Cr/C_gr_, (**a-1**,**d-1**) at the center of the interface, (**a**-**2**,**d**-**2**) corresponds to the EDS analysis results near the interface in (**a-1**,**d-1**,**a-3**,**b-2**,**c-3**,**d-3**) correspond to the magnified maps near the interface, (**b**-**3**) HRTEM of the red rectangle area in (**b-1**,**a-4**–**c-4**) correspond to the EDS analysis of lines 1–3 in (**a-3**,**b-1**,**c-3**,**d-4**) EDS analysis of points 1–9, areas 1–6 correspond to the Cu–Sn solid solution, Cr precipitates, Cu_10_Sn_3_, Cu_3_Sn, Cu_6_Sn_5_, and Sn segregation regions in contact with the RPL, the blue dotted line in (**a-3**) indicates the boundary between Cu*_x_*Sn*_y_* intermetallic compound and Cu-Sn solid solution.

**Figure 3:**
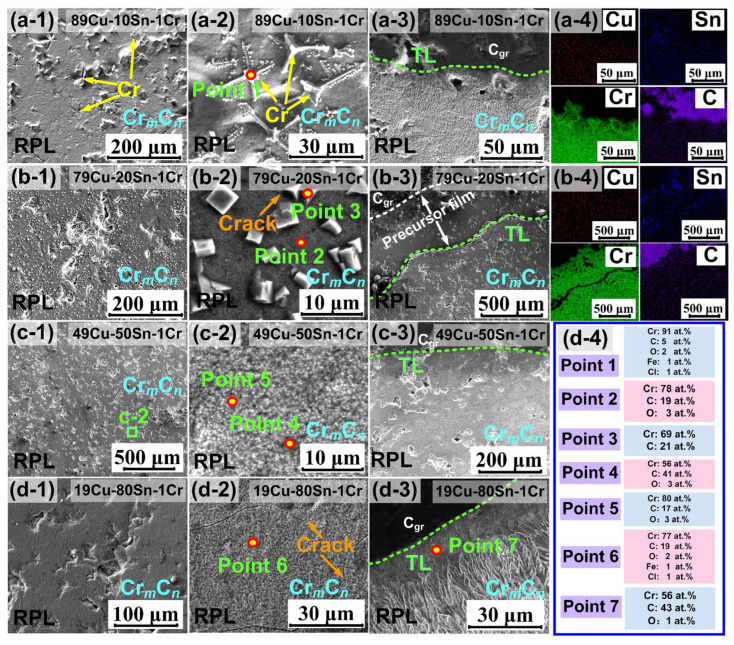
The top view SEM morphology of RPL after etching the alloy droplets (secondary electron mode): (**a-1**–**a-4**) 89Cu10Sn1Cr/C_gr_, (**b-1**–**b-4**) 79Cu–20Sn–1Cr/C_gr_, (**c-1**–**c-4**) 49Cu50Sn1Cr/C_gr_, and (**d-1**–**d-4**) 20Cu79Sn1Cr/C_gr_, (**a-1**–**d-1**) the center of the interface, (**a**-**2**–**d**-**2**) correspond to the local enlarged views of the interfaces in (**a-1**–**d-1**,**a-3**–**d-3**) local enlarged views near the TL, (**a-4**–**b-4**) correspond to the EDS analysis results of (**a-3,b-3**,**d-4**) EDS analysis results of points 1–7.

**Figure 4:**
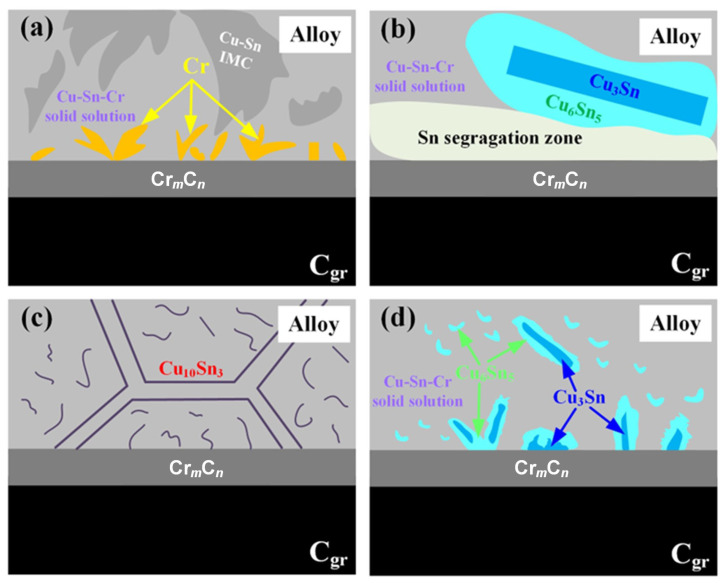
Schematic diagram of the interface structure: (**a**) Cr precipitation on the Cr*_m_*C*_n_*, surface, (**b**) Sn segregation on the Cr*_m_*C*_n_* surface, (**c**) Cu_10_Sn_3_ phase in contact with the Cr*_m_*C*_n_*, (**d**) Cu_3_Sn and Cu_6_Sn_5_ in contact with the Cr*_m_*C*_n_*.

The solidified cross-section of the Cu–*a*Sn–1Cr/C_gr_ system after wetting is examined using SEM (backscattered mode) and EDS. As shown in Figure 2(a-1–d-1), the interfacial transition zone exhibits a typical “sandwich” structure, composed of two sub-interfaces: metal||reaction product layer (RPL) and RPL||C_gr_. EDS analysis near the interface (Figure 2(a-4–c-4)) confirms that the RPL mainly consists of Cr_7_C_3_ and Cr_3_C_2_. With increasing Sn content, the dominant phase in the RPL gradually shifts from Cr_7_C_3_ (metallic) to Cr_3_C_2_ (covalent). At 10 at. % Sn (Figure 2(a-1)), Cr precipitates are observed on the RPL (point 3, Figure 2(d-4)), due to Cr’s limited solubility (0.98 at. %) in Cu–10Sn alloy at 1100 °C, which is lower than the nominal 1 at.%. As a result, a portion of Cr becomes supersaturated dissolution and precipitates out. For detailed calculations, please refer to the References [34,35,36]. When Sn reaches 20 at. %, the solubility increases to 1.96 at. %, and the Cr precipitates disappear, indicating complete dissolution into the melt (Figure 2(b-1)). The RPL becomes thicker and more continuous, increasing from ~1.4 μm (Figure 2(a-3)) to ~3 μm (Figure 2(d-3)). The alloy side exhibits various phases in contact with the RPL, including Cu–Sn solid solutions, Cu*_x_*Sn*_y_* intermetallic (e.g., Cu_10_Sn_3_, Cu_3_Sn, Cu_6_Sn_5_), and Sn/Cr segregation regions. EDS analyses in Figure 2(a-2,d-2) and points 1, 2, and 4–9 confirm that the Cu and Sn concentrations dominate, while Cr is nearly undetectable inside the alloy, justifying subsequent model simplifications. When the Sn content increases to 20 at. %, the alloy side transforms into a network structure (Figure 2(b-1)). Figure 2(b-2) shows an enlarged view near the interface of Figure 2(b-1), where the RPL thickness increases to 2.66 μm, and at the dark gray (point 4) and light gray (point 5) locations on the alloy side, Cu–Sn = 3.3:1, which may correspond to the Cu_10_Sn_3_ phase. To determine the alloy phase, FIB sampling is conducted at the location marked by the red rectangle in Figure 2(b-1). The HRTEM characterization confirms that the alloy phase is indeed Cu_10_Sn_3_, as shown in Figure 2(b-3). From Figure 2(c-1,c-2,d-1,d-3), it can be seen that Cu_3_Sn and Cu_6_Sn_5_ are the key intermetallic compounds precipitated. In addition, regions of Sn segregation may appear near the interface, as shown in Figure 2(c-3). These observations suggest multiple interfacial contact forms—Cu–Sn solid solution, Cu*_x_*Sn*_y_* (Cu_10_Sn_3_, Cu_3_Sn, and Cu_6_Sn_5_) intermetallic, Cr precipitates, and Sn segregated zones—between the alloy and RPL (Figure 2(a-1–c-3)), corresponding to areas 1–6 in Figure 2(a-1,b-1,c-1–c-3), which likely influence the IBPs, as discussed in Section 4.

Figure 3 shows the top view SEM morphology (secondary electron mode) and EDS results of the interface RPL after etching the alloy droplets using FeCl_3_ solution (mass ratio of FeCl_3_–H_2_O = 3:1). At 10 at. % Sn, dendritic Cr-rich precipitates appear in the RPL (Figure 3(a-1,a-2)), confirmed by EDS (point 1, Figure 3(d-4)) as mainly Cr with traces of C, O, Fe, and Cl—the latter likely from residual etchant. Figure 3(a-3–d-3) show the morphology near the triple line (TL). EDS analysis in Figure 3(a-4,b-4) indicates that the RPL is primarily composed of Cr, with small amounts of Cu, Sn, and C. The precipitates on the RPL are mainly concentrated at the center of the interface, with fewer near the TL. As the Sn content increases to 20 at. %, the dendritic Cr-rich phases disappear, replaced by numerous polygonal Cr*_m_*C*_n_* phases (Figure 3(b-1)). EDS analysis of point 3 suggests that the particles (point 3) in Figure 3(b-2) have a composition similar to that of the point 2 and may be the Cr_7_C_3_ phase. A precursor film enriched in Cr and C elements ahead of the RPL (Figure 3(b-3,b-4)), possibly due to Sn-induced Cr migration toward the TL. With 50 at. % Sn, no precipitates are seen on the RPL (Figure 3(c-1)). The RPL consists of nanoparticles in orthorhombic Cr_3_C_2_ (point 4) and hexagonal Cr_7_C_3_ (point 5) phases, as shown in Figure 3(c-1,c-2). At 80 at. % Sn, there are some cracks distributed in the continuous and dense Cr*_m_*C*_n_* layer (Figure 3(d-2)), possibly caused by stress concentration during cooling. EDS of point 6 suggests central RPL regions are Cr_7_C_3_, while river-like structures at the TL front (point 7) show Cr/C atomic ratios close to Cr_3_C_2_. These results indicate that Sn content not only alters the RPL morphology but also affects Cr diffusion and interfacial phase distribution. Further phase confirmation can be found in the X-ray diffraction results in Ref. [14].

## 4. Discussion

From the above, the interface structure analysis indicates that the Cu–*a*Sn–1Cr/C_gr_ reactive wetting system exhibits a “sandwich” structure, composed of two interfaces: metal||Cr*_m_*C*_n_* and Cr*_m_*C*_n_*||C_gr_. For the latter, although this study does not directly investigate the bonding mechanism at the Cr*_m_*C*_n_*||C_gr_ interface, previous work (Ref. [37]) has reported that bonding at the Cr_3_C_2_||diamond interface primarily arises from hybridization between C–2*p* states of diamond (001) and C–2*p* or Cr–3*d* states of Cr_3_C_2_ (001). Given the structural and electronic similarity between diamond and C_gr_, it is reasonable to expect a similar bonding mechanism at the Cr*_m_*C*_n_*||C_gr_ interface in our system. Therefore, detailed discussion of this interface is omitted here. In this work, we focus on the metal||Cr*_m_*C*_n_* interface, as it plays a more dominant role in determining the overall wettability behavior observed in our experiments. As illustrated in Figure 4, Sn and Cr may interact with the RPL surface in multiple forms, including Cu–Sn solid solutions, Cu*_x_*Sn*_y_* compounds, and Sn/Cr segregation.

From Section 3.2, it can be seen that when the Sn content is low, Cr undergoes supersaturation and precipitates out, and when the Sn content is high, according to the Cu–Sn binary phase diagram [38], Sn segregation occurs, and Cu*_x_*Sn*_y_* varies with the Sn content. The contact phenomenon with the RPL surface may be caused by the strong interactions among Sn/Cr and Cu*_x_*Sn*_y_* with Cr*_m_*C*_n_*. The EDS analysis in Figure 2 of Section 3.2 shows that the main precipitate phases in contact with RPL are Cu_10_Sn_3_, Cu_3_Sn, and Cu_6_Sn_5_, which may result from reaction formulas Equations (3)–(10) occurring during the cooling process:

10Cu + 3Sn → Cu_10_Sn_3_ (3)

3Cu + Sn → Cu_3_Sn (4)

Cu + 5Sn → Cu_6_Sn_5_ (5)

Cu_10_Sn_3_→ Cu + 3Cu_3_Sn (6)

Cu_6_Sn_5_ + 9Cu → 5Cu_3_Sn (7)

5Cu_3_Sn → 9Cu + Cu_6_Sn_5_ (8)

2Cu_3_Sn + 3Sn → Cu_6_Sn_5_ (9)

L + Cu_3_Sn → Cu_6_Sn_5_ (10)

Li et al. [39] reported that adding 1~2 wt. % Cr does not significantly affect the growth of Cu*_x_*Sn*_y_*. In the initial reaction stage, Cu_10_Sn_3_, Cu_3_Sn, and Cu_6_Sn_5_ are formed (Equations (3)–(5)), where Cu_10_Sn_3_ is a metastable phase that readily transforms into Cu_3_Sn (Equation (6)). When there is less Sn, Cu_6_Sn_5_ may convert to Cu_3_Sn (Equation (7)). Conversely, when there is more Sn, Cu_3_Sn will transform into Cu_6_Sn_5_, and Cu_3_Sn will decompose to form Cu_6_Sn_5_ (Equation (8)). Sn atoms continuously diffuse into Cu_3_Sn and also react with Sn to produce Cu_6_Sn_5_ (Equation (9)). Furthermore, the outer layer of Cu_3_Sn can also transform into Cu_6_Sn_5_ through a peritectic reaction (Equation (10)) [40]. To investigate the effect of the aforementioned forms of metal presence on the interfacial properties and to illustrate the specific mechanism of alloying elements, this paper focuses on the metal||Cr*_m_*C*_n_* interface. The electronic structure calculations of Cr_7_C_3_ and Cr_3_C_2_ indicate that the chemical bonds in these two compounds exhibit complex characteristics with a mixture of metallic, covalent, and ionic properties [41,42]. The difference between the two was primarily reflected in the stronger metallic of Cr_7_C_3_ than that of Cr_3_C_2_. Naidich [11] reported that the metallic properties of ceramics enhance the metal/ceramic adhesion. However, Cr_3_C_2_ is more stable than Cr_7_C_3_. Considering the computational cost (Cr_7_C_3_ requires more atoms for modeling than Cr_3_C_2_), the most stable Cr_3_C_2_ is chosen for this study. Based on the interface structure obtained from the experiments, this study approximates several typical configurations at the metal||Cr_3_C_2_ interface as follows: the effects of Cu–Cr and Cu–Sn solid solutions are simplified to the issue of Cr/Sn mono-/multi-atomic doping in the Cu matrix. The precipitation of Cr/Sn on the interface is simplified to the issue of contact between pure Cr/Sn and Cr_3_C_2_. The case with high Sn content is simplified to the influence of Cu*_x_*Sn*_y_* (the effect of Cu*_x_*Sn*_y_* on the interface also needs to be explored). Simplified interface models are established for pure metals (Cu/Sn/Cr), Cu-doped Cr/Sn single/multi-atom systems, and Cu*_x_*Sn*_y_* (Cu_10_Sn_3_, Cu_3_Sn, Cu_6_Sn_5_) with Cr_3_C_2_.

### 4.1. DFT Calculations

#### 4.1.1. Surface Properties

The lattice parameters of Cu, *β*–Sn, Cr, Cu*_x_*Sn*_y_* (Cu_10_Sn_3_, Cu_3_Sn, Cu_6_Sn_5_), and Cr_3_C_2_ are shown in Table S1 in the Supplementary Materials. The optimized lattice constant values are compared with the calculated values from References [37,43,44,45,46], and there is a slight deviation of ± 2% in the obtained lattice parameters. The close-packed surfaces of Cu (111), *β*–Sn (100), Cr (110), and Cr_3_C_2_ (001) have stable atomic structures and the lowest surface energies [37,47,48,49], and the surface models are shown in Figure S2a–d. Here, the reason for selecting the surface with the lowest surface energy and high adhesion work for the calculation is explained below. The atomic radius difference between Cu and Sn atoms is large, and the lattice distortion is small when the doping quantity is low. Conversely, at higher doping quantities, the lattice distortion is larger. Therefore, to investigate the effect of high Sn content, this paper uses the Cu*_x_*Sn*_y_* compounds shown in Figure 2(b-2,c-1,c-2) of Section 3.2 for comparison. The surface energy of Cu*_x_*Sn*_y_* is investigated and found to be rarely reported in the literature, so the surface energy of Cu*_x_*Sn*_y_* is calculated before building the interface model. According to Ref. [50], it is reported that the maximum Miller indices are 2 and 3 for noncubic and cubic crystals, respectively, when calculating the surface energy. Due to the limitation of the calculation conditions, in this paper, we only investigate the surface properties of (100), (010), and (001) of Cu_10_Sn_3_, Cu_3_Sn, and Cu_6_Sn_5_. Based on the symmetry and periodicity of the structures, surfaces with the same structure but different indices are combined for analysis, and the constructed surface structure models are presented in Supplementary Materials Figure S2e–j. *σ*_metal_ is commonly defined as the energy required per unit area to form a new surface, which can be used to describe the stability of the surface, and is given by Equation (11) [51]:

(11) $$\sigma_{\mathrm{metal}}=\frac{E_{\mathrm{surface}}-{nE}_{\mathrm{bulk}}-{n_{i}\mu }_{i}+n_{j}\mu_{j}}{2A_{\mathrm{surface}}}$$

**Figure 5:**
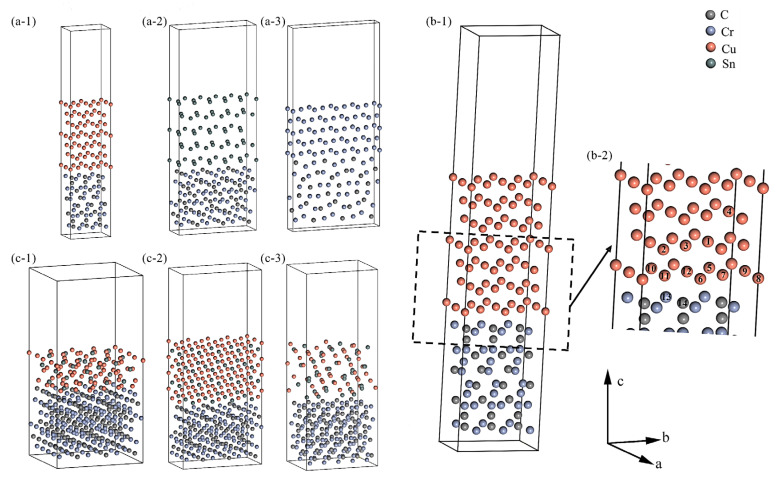
Interface model: (**a-1**–**a-3**) pure metal Cu/Sn/Cr with Cr_3_C_2_: (**a-1**) Cu||Cr_3_C_2_, (**a-2**) Sn||Cr_3_C_2_, (**a-3**) Cr||Cr_3_C_2_, (**b-1**) Cr/Sn atom doped regions, (**b-2**) doping positions and their numbers, Numbers 1–12 indicate the atomic positions for Cr atom doping in the Cu||Cr_3_C_2_ interface, Numbers 1–14 represent the positions for Sn atom doping in the same interface. (**c-1**–**c-3**) Cu*_x_*Sn*_y_*||Cr_3_C_2_: (**c-1**) Cu_10_Sn_3_||Cr_3_C_2_, (**c-2**) Cu_3_Sn||Cr_3_C_2_, (**c-3**) Cu_6_Sn_5_||Cr_3_C_2_.

*σ*_metal_ is the surface energy of the metal (J/m^2^); *E*_surface_, *n, E*_bulk_, and *A*_surface_ are the total energy of the metal surface, the number of unit cells, the energy of the unit cell, and the area of the surface, respectively. The doped surface model also needs to consider the number of dopant atoms and their chemical potential, where *n_i_* and *n_j_* are the number of dopant atoms and doped atoms, respectively. The *μ_i_* and *μ_j_* are the chemical potential of the dopant atoms and doped atoms, respectively. The *σ*_metal_ values for Cu (111), Sn (100), Cr (110), Cu_6_Sn_5_ (100), and Cr_3_C_2_ (001) are close to the reported results in the References [37,45,52,53,54], as presented in Table S2 in Supplementary Materials. The doping cases are as follows: single Cr atom doped Cu (111) and Sn (100) surfaces, named Cu_55_Cr_1_ and Sn_49_Cr_1_, respectively. Multiple Sn atoms doped Cu (111) surface with up to four dopant atoms, named Cu_56-*k*_Sn*_k_* (*k* = 1, 2, 3, 4), the doping position is shown in Figure 5(b-2). As shown in Table S2, the doping of a small number of Cr atoms increases the *σ*_metal_ of Cu (111) but has little effect on the *σ*_metal_ of Sn (100). When multiple Sn atoms are doped, the *σ* (Cu_56-*k*_Sn*_k_*) decreases slightly with increasing Sn atoms, while the *σ*_metal_ of Cu*_x_*Sn*_y_* decreases significantly with the increase in Sn content. This trend is consistent with the changes in *σ*_lv_ of Cu–Sn alloys reported in Ref. [29]. Cu_10_Sn_3_ (100), Cu_3_Sn (001) type2, and Cu_6_Sn_5_ (001) type2 with lower *σ*_metal_ values are selected for the study in the case of higher Sn content.

#### 4.1.2. Interfacial Details

Before establishing the interface models of pure metals (Cu/Sn/Cr), Cu-doped single/multiple Cr/Sn atoms, and Cu*_x_*Sn*_y_* with Cr_3_C_2_, the mismatch degree *δ* of the interfaces is calculated. Ref. [55] shows that when *δ* < 8%, the two surfaces can be considered well-matched, which allows the interface model to achieve good convergence. The formula for calculating *δ* is shown in Equation (12) [56]:

(12) $$\begin{array}{c} \delta =\frac{\left|a_{1}\;-\;a_{2}\right|}{0.5{(a}_{1}+a_{2})}\times 100\% \end{array}$$

where *a*_1_ and *a*_2_ (Å) represent the lengths of the supercells that need to be matched in the same direction. $\left|a_{1}-a_{2}\right|$ is the difference between the matched lattice parameters, and $0.5{\;(a}_{1}+a_{2})$ is their average. The *δ*–values for the supercell interface models are all below 8%, which satisfies the matching criteria. The calculated *δ*–values are summarized in Table S3.

Based on this, interface models are constructed for Cu (111)||Cr_3_C_2_ (001), Sn (100)||Cr_3_C_2_ (001), Cr (110)||Cr_3_C_2_ (001), and the three compound interfaces Cu_10_Sn_3_ (100), Cu_3_Sn (001), and Cu_6_Sn_5_ (001) with Cr_3_C_2_ (001). These are denoted as Cu||Cr_3_C_2_, Sn||Cr_3_C_2_, Cr||Cr_3_C_2_, and Cu*_x_*Sn*_y_*||Cr_3_C_2_ (Cu*_x_*Sn*_y_* = Cu_10_Sn_3_, Cu_3_Sn, and Cu_6_Sn_5_), respectively, and are shown in Figure 5(a-1–a-3,c-1–c-3). A vacuum layer of 15 Å is added along the *c*-axis in all interface models to eliminate the effect of periodic boundary conditions.

To study the effect of atomic doping, 14 and 12 possible substitution positions are considered for single Cr or Sn atoms in Cu||Cr_3_C_2_ and Sn||Cr_3_C_2_ models, respectively, as shown in Figure 5(b-2). The doping difficulty at each site is evaluated by calculating the formation energy *E*_sub_ [57] (details in Supplementary Materials). The configurations with the lowest *E*_sub_ are selected and denoted as Cu_55_Cr_1_||Cr_3_C_2_ and Sn_49_Cr_1_||Cr_3_C_2_. For multiple Sn atom doping, four low *E*_sub_ positions are selected, labeled as Cu_56-*k*_Sn*_k_*||Cr_3_C_2_ (*k* = 1, 2, 3, 4).

#### 4.1.3. Interface Properties

The adhesion, stability, and wettability in the interfacial properties are related to the adhesion work and the surface/interfacial energy of the metal and Cr_3_C_2_. Therefore, this section primarily analyzes the effects of typical interface structures on interfacial properties at the atomic level by calculating the theoretical adhesion work *W*_ad_ and the interfacial energy *γ*_int_. *W*_ad_ is obtained by Equation (13) [55]:

(13) $$W_{\mathrm{ad}}=\frac{E_{\mathrm{metal}}+E_{Cr_{3}C_{2}}-E_{\mathrm{int}}}{A_{\mathrm{int}}}$$

where $E_{\mathrm{metal}}$ is the energy of the metal part, $E_{Cr_{3}C_{2}}$ is the energy of Cr_3_C_2_, *E*_int_ is the total energy of the interface model, and *A*_int_ is the area of the interface. When *W*_ad_ is larger, the interface is more stable and the interfacial bond strength is higher. The interfacial equilibrium distance of the interface is determined based on the maximum value of the *W*_ad_, as detailed in Figure S3 in Supplementary Materials. The *γ*_int_ is calculated as shown in Equation (14) [55]:

(14) $$\gamma_{\mathrm{int}}=\sigma_{\mathrm{metal}}+\sigma_{{\mathrm{Cr}}_{3}C_{2}}-W_{\mathrm{ad}}$$

where *σ*_Cr_3_C_2__ is the surface energy of the Cr_3_C_2_. A lower *γ*_int_ indicates a more stable interface, as wetting is preferred to be carried out at interfaces with lower values of *γ*_int_, which is often used as a basis for determining model parameters. The minimum value of *σ*_Cr_3_C_2__ (001) is 3.99 J/m^2^. *σ*_metal_ and *W*_ad_ vary with the composition of the metal. For a stable interface to form between *σ*_metal_ and *σ*_Cr_3_C_2__, *γ*_int_ must be positive. If *γ*_int_ is negative, the interface is unstable. The lower the *γ*_int_ value (closer to zero), the more stable the interface. To ensure the value of *γ*_int_ is the minimum, the surface/interface models with the smallest difference of (*σ*_metal_ − *W*_ad_) are chosen for calculation as much as possible. However, selecting a surface/interface model with a minimum (*σ*_metal_ − *W*_ad_), *γ*_int_ > 0, and low *δ* requires extensive calculations. Therefore, the calculations in this paper utilize a maximum *W*_ad_ and a relatively low *σ*_metal_.

(1) Single Sn/Cr atom and multiple Sn atoms doped Cu||Cr_3_C_2_ interface

**Figure 6:**
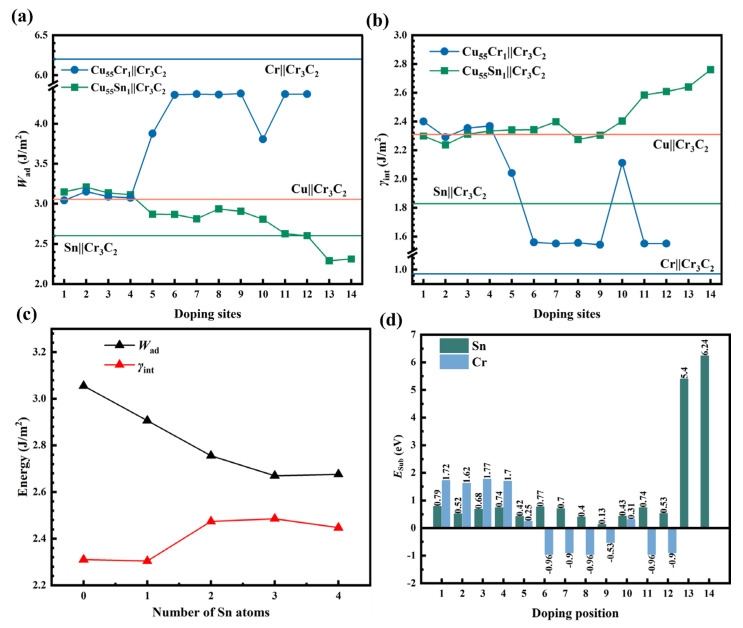
The variation of *W*_ad_ and *γ*_int_ when a single Sn/Cr atom and multiple Sn atoms are doped at interface of the Cu||Cr_3_C_2_ interface and the magnitude of *E*_sub_: (**a**) the *W*_ad_ of Sn/Cr atom dope into different sites at the Cu||Cr_3_C_2_ interface; (**b**) *γ*_int_, the red, green, and blue horizontal lines correspond to Cu||Cr_3_C_2_, Sn||Cr_3_C_2_ and Cr||Cr_3_C_2_, respectively; (**c**) *W*_ad_ and *γ*_int_ for the Cu_56-*k*_Sn*_k_*||Cr_3_C_2_ (*k* = 0, 1, 2, 3, 4) interface; (**d**) the magnitude of *E*_sub_.

Figure 6 shows the variation of *W*_ad_ (Figure 6a) and *γ*_int_ (Figure 6b) for a single Sn/Cr atom doping at different positions of Cu||Cr_3_C_2_. From Figure 6a, it can be seen that the magnitude of *W*_ad_ is in the order of Sn||Cr_3_C_2_ (2.60 J/m^2^) < Cu||Cr_3_C_2_ (3.05 J/m^2^) < Cr||Cr_3_C_2_ (6.20 J/m^2^). This suggests that the precipitation of Cr on RPL, as shown in Figure 3(a-2) of Section 3.2, plays a role in enhancing the interface bonding. The *W*_ad_ of Cu||Cr_3_C_2_ is slightly higher than the value of *W*_ad_ (2.16 J/m^2^) in Ref [33]. Comparing the *W*_ad_ of Cu_55_Cr_1_||Cr_3_C_2_ and Cu_55_Sn_1_||Cr_3_C_2_, it is clear that the influence of Sn/Cr on the *W*_ad_ at positions 5–12 of the Cu||Cr_3_C_2_ is much greater than that at positions 1–4. Thus, the further the doping site is from the Cr_3_C_2_ surface, the smaller the effect. As shown in Figure 6a, except for positions 1–4, Sn doping at other sites decreases the *W*_ad_ of the interface. From Figure 6b, it can be seen that the Cr||Cr_3_C_2_ is more stable and possesses the lower *γ*_int_ (0.97 J/m^2^) compared to Cu||Cr_3_C_2_(2.31 J/m^2^) and Sn||Cr_3_C_2_(1.83 J/m^2^). Different Sn doping sites have less effect on the *γ*_int_ of Cu||Cr_3_C_2_, whereas Cr doping significantly reduces the *γ*_int_ of Cu||Cr_3_C_2_ and enhances the stability of the interface. The *W*_ad_ and *γ*_int_ at sites 13 and 14 in Figure 6 correspond to Sn atoms occupying the Cr and C sites in Cr_3_C_2_. It can be seen that the doping at these two sites significantly decreases the *W*_ad_ of the Cu||Cr_3_C_2_ and increases the *γ*_int_, resulting in the interface being unstable. Therefore, the possibility of Sn occupying sites in Cr_3_C_2_ is relatively low. In addition, the *W*_ad_ of Sn_49_Cr_1_||Cr_3_C_2_ (3.99 J/m^2^) is higher than that of Sn||Cr_3_C_2_ (2.60 J/m^2^), and the *γ*_int_ of Sn_49_Cr_1_||Cr_3_C_2_ (0.84 J/m^2^) is lower than that of Sn||Cr_3_C_2_ (1.83 J/m^2^), indicating a significant improvement in interface stability.

(2) Cu_x_Sn_y_||Cr_3_C_2_ interface

After being compared to Cu||Cr_3_C_2_, Sn||Cr_3_C_2_, Cr||Cr_3_C_2_, and Cu_56-*k*_Sn*_k_*||Cr_3_C_2_, the Cu_10_Sn_3_||Cr_3_C_2_ (*W_ad_* = 1.28 J/m^2^, *γ*_int_ = 3.58 J/m^2^), Cu_3_Sn||Cr_3_C_2_ (*W_ad_* = 1.09 J/m^2^, *γ*_int_ = 3.93 J/m^2^), and Cu_6_Sn_5_||Cr_3_C_2_ (*W_ad_* = 0.96 J/m^2^, *γ*_int_ = 3.79 J/m^2^) interface possesses lower *W*_ad_ and higher *γ*_int_. The *W*_ad_ gradually decreases, with increasing Sn content in Cu*_x_*Sn*_y_* increases. Although the *γ*_int_ fluctuates, they are overall relatively high, which indicates that the interface between Cu*_x_*Sn*_y_* and Cr_3_C_2_ is unstable. However, once Cu*_x_*Sn*_y_* interacts with Cr_3_C_2_ to form a Cu*_x_*Sn*_y_*||Cr_3_C_2_ interface structure, it will be unfavorable for interfacial bonding. Yu et al. [10] studied the interfacial properties of Cu*_x_*Sn*_y_* with diamond and the results indicated that Cu_6_Sn_5_ (100) with diamond (111) had a stronger hybridization, lower *γ*_int_, and better wettability compared to Cu (100) and Cu_3_Sn (100). Cu*_x_*Sn*_y_* had different effects on the Cu*_x_*Sn*_y_*||diamond and Cu*_x_*Sn*_y_*||Cr_3_C_2_ interfaces. Therefore, when considering the adjustment of Sn content to improve the wettability of Cu–*a*Sn–1Cr/C_gr_ or enhance the mechanical and functional properties of the Cu matrix, the effects of Cu*_x_*Sn*_y_* on *W*_ad_ and *γ*_int_ should also be taken into account. This provides theoretical guidance for the design and property optimization of compositions in Cu–*a*Sn–1Cr/C_gr_ and similar systems.

(3) Comparison of theoretical and calculated values

Table 1 has the $W_{\mathrm{ad}}^{\mathrm{cal}}$ obtained in Section 3.1 from experimental values of *θ*_e_, *σ*_lv_ [29] and solid–liquid interfacial energy *σ*_sl_; *σ*_sl_ can be obtained from Equation (15) [58,59]:

(15) $$\cos\theta_{e}=\frac{\sigma_{\mathrm{sv}}-\sigma_{\mathrm{sl}}}{\sigma_{\mathrm{lv}}}$$

The value of *σ*_sv_ of Cr_3_C_2_ is 4.13 J/m^2^ [37]. The *a* (at. %) values are obtained by converting the Sn content in the metal surface model according to the atomic ratio. Similarly, the *σ*_metal_, *W*_ad_, and *γ*_int_ obtained from the theoretical calculations are displayed in Table 2. Comparing Table 1 and Table 2, although there is a certain deviation between the theoretically calculated values and the experimentally calculated values, there is a trend of decreasing *σ*_metal_ and *σ*_lv_, as well as *W*_ad_ and $W_{\mathrm{ad}}^{\mathrm{cal}}$ with the increase in *a*. The values of *σ*_metal_ and *σ*_lv_ are close to each other. However, some theoretically calculated values (*W*_ad_ and *γ*_int_) are larger than the experimentally calculated values ($W_{\mathrm{ad}}^{\mathrm{cal}}$ and *σ*_sl_), such as the Cu||Cr_3_C_2_ and Sn||Cr_3_C_2_ interfaces, which results in an inability to back extrapolate *θ*_e_ and thus hinders wettability predictions. The reason may be that *W*_ad_ and *γ*_int_ are based on first-principles calculations at 0 K, where the atoms are in equilibrium positions, whereas the experimentally calculated values are obtained from measurement and calculation after heating to 1100 °C. The theoretically calculated values of *W*_ad_ are smaller than $W_{\mathrm{ad}}^{\mathrm{cal}}$, and *γ*_int_ is larger than *σ*_sl_ for Cu_56-*k*_Sn*_k_*||Cr_3_C_2_ and Cu*_x_*Sn*_y_*||Cr_3_C_2_, probably because the composition of the RPL in the experiment includes not only Cr_3_C_2_ but also Cr_7_C_3_, which affects the experimental values and hence the larger values.

#### 4.1.4. Interfacial Electronic Structure

The bonding of interface atoms depends on the configuration of the metal||Cr*_m_*C*_n_* interface. To better explore the interactions between atoms at the metal||Cr*_m_*C*_n_* interface, this section calculates the changes in the partial density of states (PDOS), charge density difference (CDD), and Mulliken population of the interface after relaxation and reveals the variation mechanism of the IBPs from an electronic perspective (charge transfer and distribution).

(1) Partial density of states

**Figure 7:**
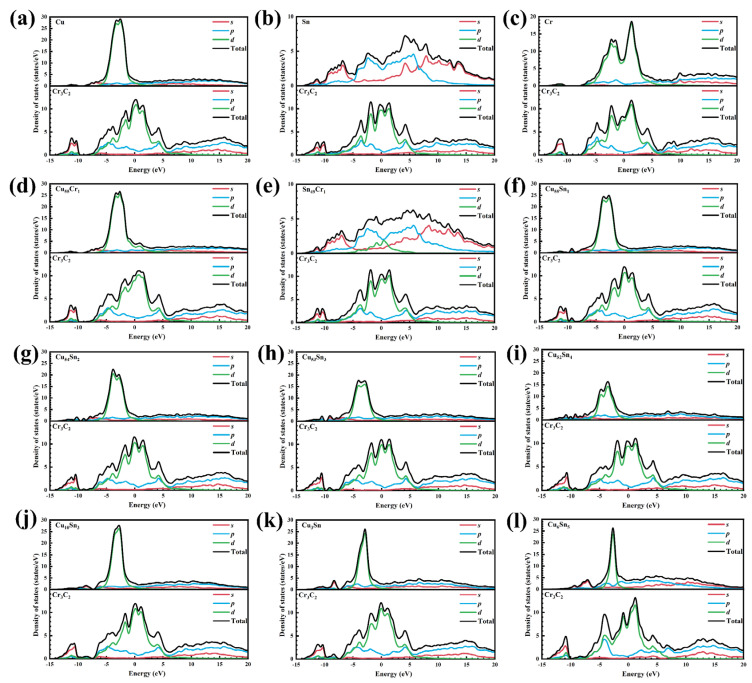
Partial density of states (PDOS) at interfaces: (**a**) Cu||Cr_3_C_2_, (**b**) Sn||Cr_3_C_2_, (**c**) Cr||Cr_3_C_2_, (**d**) Cu_54_Cr_1_||Cr_3_C_2_, (**e**) Sn_49_Cr_1_||Cr_3_C_2_, (**f**–**i**) Cu_56-*k*_Sn*_k_*||Cr_3_C_2_: (**f**) *k* = 1, (**g**) *k* = 2, (**h**) *k* = 3, (**i**) *k* = 4, (**j**–**l**) Cu*_x_*Sn*_y_*||Cr_3_C_2_: (**j**) Cu_10_Sn_3_||Cr_3_C_2_, (**k**) Cu_3_Sn||Cr_3_C_2_, (**l**) Cu_6_Sn_5_||Cr_3_C_2_.

Figure 7 shows the PDOS of the above interfacial structures near the Fermi energy level (E_F_). The PDOS of the electronic orbitals of the relaxation state system can be used to determine the origin of the electronic orbitals that contribute to interatomic interactions in each system. In Figure 7, the upper and lower parts of each subplot represent the PDOS of the metal and the Cr_3_C_2_, respectively. The PDOS is divided into *s*, *p*, and *d* orbitals based on the orbitals in which the valence electrons are located, and the total is the sum of all orbitals of the metal||Cr_3_C_2_ part. Figure 7a–c correspond to the PDOS for the interfaces Cu||Cr_3_C_2_, Sn||Cr_3_C_2_, and Cr||Cr_3_C_2_, respectively. In the Cu||Cr_3_C_2_ interface (Figure 7a), the main contributions stem from the electronic interactions of the Cr–*d* and Cu–*d* orbitals. The density of states (DOS) of the *d* orbital electrons of Cu and Cr_3_C_2_ are mainly located in the range of −6eV~−0.9eV and −7.5eV~5.5eV. The range of DOS distribution of the *d* orbital electrons of Cr_3_C_2_ is generally consistent with that reported in Ref. [37]. Similarly, for the Cr||Cr_3_C_2_ interface, the *d* orbital contributes to the main electronic state (Figure 7c), where the Cr–*d* orbital of Cr hybridizes with the Cr–*d* orbital of Cr_3_C_2_. The metallic bonds formed at the interface of Cu||Cr_3_C_2_ and Cr||Cr_3_C_2_ provide the main role for adhesion at the interface. The DOS of Sn is mainly contributed by the Sn–*p* orbital electrons (Figure 7b), which are mainly distributed in the range of −5eV~9eV, with a small contribution from the *d* orbital electrons. The Sn||Cr_3_C_2_ interface is mainly contributed by metallic bonds formed by Sn–*p* orbital and Cr–*d* orbital electrons. The bonding strength Cr||Cr_3_C_2_ > Cu||Cr_3_C_2_ > Sn||Cr_3_C_2_ is due to the metallic bonds formed by *p*–*d* orbital hybridization being weaker than those formed by *d*–*d* orbital hybridization, and thus Sn||Cr_3_C_2_ is lower. Comparing Figure 7a,c, the DOS peak for Cr||Cr_3_C_2_ shows *d*–*d* orbital hybridization over a wider energy range, which has more homomorphic electrons compared to Cu||Cr_3_C_2_, and thus Cr||Cr_3_C_2_ is higher. Comparing Figure 7a,d, it is observed that the DOS near the E_F_ for the Cu_55_Cr_1_||Cr_3_C_2_ interface is higher than that of Cu||Cr_3_C_2_. This is because Cr doping effectively increases the *d* orbital electronics near the E_F_ at the Cu||Cr_3_C_2_ interface, thereby enhancing the bonding strength between the interface atoms. Comparing Figure 7a,d, it is observed that the DOS of *d* orbital electronics below the E_F_ for Cu_55_Cr_1_ is higher than for Cu. Similarly, Figure 7b,e show that Sn_49_Cr_1_ increases the DOS of *d* orbital electronics below the E_F_ for Sn. Therefore, Cr doping can effectively enhance the interface bonding strength. Figure 7f–i show the effect of doping with different numbers of Sn atoms on the PDOS near the E_F_ at the interface. Comparing Figure 7a,f, it is evident that Sn doping causes the peak value of the DOS for the *d* orbital electrons of Cu to decrease from 28 eV/states to 25 eV/states. With the increase in Sn doping number *k* (*k* = 1, 2, 3, 4), the DOS peak of the *d* orbital electrons below the E_F_ in the Cu_56-*k*_Sn*_k_* decreases gradually, which indicates that Sn doping weakens the interfacial bonding strength. Figure 7j–l shows the effect of Cu*_x_*Sn*_y_* on PDOS near the E_F_ at the interface. With the increase in Sn content, the DOS peak widths of the *d* orbital electrons below the E_F_ of Cu*_x_*Sn*_y_* are in the order of Cu_10_Sn_3_ > Cu_3_Sn > Cu_6_Sn_5_. Compared with Figure 7f–i, the DOS peak widths of the *d* orbital electrons below the E_F_ of Cu*_x_*Sn*_y_* are narrower than those of Cu_56-*k*_Sn*_k_*, which indicates that higher Sn content results in the number of *d* orbital electrons of Cu*_x_*Sn*_y_* being less than that of Cu_56-*k*_Sn*_k_*, so the bonding strength of the interface is gradually weakened.

(2) Charge density difference analysis

**Figure 8:**
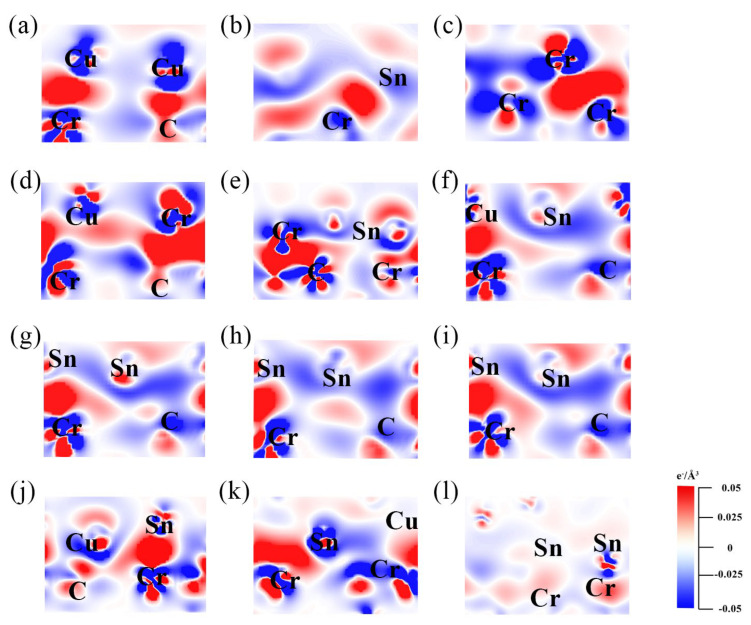
Charge density difference (CDD) maps of selected metal||Cr_3_C_2_ interfaces; red and blue regions represent charge accumulation and dissipation, respectively: (**a**) Cu||Cr_3_C_2_, (**b**) Sn||Cr_3_C_2_, (**c**) Cr||Cr_3_C_2_, (**d**) Cu_54_Cr_1_||Cr_3_C_2_, (**e**) Sn_49_Cr_1_||Cr_3_C_2_, (**f**–**i**) Cu_56-*k*_Sn*_k_*|| Cr_3_C_2_: (**f**) *k* = 1, (**g**) *k* = 2, (**h**) *k* = 3, (**i**) *k* = 4, (**j**–**l**) Cu*_x_*Sn*_y_*||Cr_3_C_2_: (**j**) Cu_10_Sn_3_||Cr_3_C_2_, (**k**) Cu_3_Sn||Cr_3_C_2_, (**l**) Cu_6_Sn_5_||Cr_3_C_2_.

To further explore the interfacial bonding mechanisms at the atomic scale, charge density difference (CDD) analyses are performed for typical metal||Cr_3_C_2_ interfaces with different alloying configurations. The CDD (Δ*ρ*) is used to indicate the charge density redistribution and the degree of electron transfer at the interface. The Δ*ρ* can be expressed as Equation (16) [52]:

(16) $$\Delta \rho \;={\;\rho }_{\mathrm{int}}-\rho_{\mathrm{metal}}-{\;\rho }_{{\mathrm{Cr}}_{3}C_{2}}$$

where *ρ*_int_ is the total charge density of the metal||Cr_3_C_2_ interface, *ρ*_metal_, and *ρ*_Cr3C2_ are the charge densities of isolated metal slab and Cr_3_C_2_ slab, respectively. The red regions represent charge accumulation (electron gain), while the blue regions indicate charge dissipation (electron loss). The intensity of the color corresponds to the degree of charge accumulation or dissipation. The CDD results (Figure 8) show significant charge accumulation and dissipation at the Cr||Cr_3_C_2_ interface compared to Cu||Cr_3_C_2_ and Sn||Cr_3_C_2_, indicating strong interfacial charge transfer and bond formation, which is consistent with its high adhesion work (*W*_ad_). In addition, the Cr-doped interfaces (Cu_55_Cr_1_||Cr_3_C_2_ and Sn_49_Cr_1_||Cr_3_C_2_) exhibit enhanced charge redistribution, confirming that Cr doping strengthens interfacial bonding. As the Sn doping content increases (Cu_56_−_*k*_Sn_*k*_||Cr_3_C_2_, *k* = 1–4), the charge accumulation near the interface gradually weakens, and dissipation becomes more prominent, as shown in Figure 8f–i. This trend continues in the Cu*_x_*Sn*_y_*||Cr_3_C_2_ interfaces, as shown in Figure 8j–l, particularly at Cu_6_Sn_5_||Cr_3_C_2_, where charge dissipation is strongest, corresponding to the lowest *W*_ad_ observed.

## 5. Conclusions

In the reactive wetting experiments of the Cu–*a*Sn–1Cr/C_gr_ system, variations in the Sn content can affect the physical and chemical properties of the alloy, the dissolution/precipitation of Cr elements, the microstructure of the alloy, and the phase composition and morphology of the RPL. These changes further impact the interface configurations and characteristics. Based on the first-principles study, several typical interfacial structures are selected, and simplified interfacial models are constructed. By calculating the *W*_ad_, *σ*_metal_, and *γ*_int_ of these models at the atomic level, the effects of these factors on the interface characteristics are elucidated. Additionally, from the electronic level, the nature of the effects of interatomic charge distribution on interfacial properties is explored using the PDOS and CDD. The conclusions from the experiments and calculations are as follows:

(1) With the increase in Sn content, the *σ*_lv_ and $W_{\mathrm{ad}}^{\mathrm{cal}}$ of the alloy show a decreasing trend, and the RPL thickness increases. The overall interface presents a “sandwich” structure, consisting of the metal||Cr*_m_*C*_n_* (Cr_3_C_2_ and Cr_7_C_3_) and Cr*_m_*C*_n_*||C_gr_ interfaces. The influence of varying Sn content is primarily reflected in the metal||Cr*_m_*C*_n_* interface. The phase composition and morphology of Cr*_m_*C*_n_* change with the Sn content.
(2) The various forms (Cu–Sn solid solutions, Cu*_x_*Sn*_y_* compounds, and Sn/Cr element segregation) exist on the alloy side in contact with the Cr*_m_*C*_n_* surface. The doping and segregation of Cr can significantly increase the *W*_ad_ and stability of the metal||Cr_3_C_2_ interface. The effect of Sn is opposite to that of Cr and becomes more significant with the increase in Sn content. This is primarily because the metallic bond formed by Sn–*p* and Cr–*d* is weaker than the *d*–*d* metallic bonds (Cu–*d* and Cr–*d*, Cr–*d* and Cr–*d*). Sn increases the dissipation (or accumulation) of the interface’s CDD. The increase in Sn content reduces the number of *d* orbital electrons in the metal portion, leading to a decrease in the PDOS of electronics below the E_F_.
(3) The *σ*_metal_ of the Cu–Sn alloy obtained from theoretical calculations is in good agreement with the *σ*_lv_ reported in the literature. The trend in the theoretical calculation of *W*_ad_ agrees with that of $W_{\mathrm{ad}}^{\mathrm{cal}}$ calculated in the experiment.
(4) The systematic experiments and simplified interfacial models based on the first-principles DFT calculations help elucidate the interaction among alloying elements and the effects of Sn content changes; reveal how typical interfacial configurations affect wettability, adhesion, and stability; and reduce the need for extensive experimental screening to avoid the influence of complex experimental conditions. This combined approach provides useful insights for designing interfaces in metal/carbon composites. In future work, advanced characterization techniques coupled with multiscale and dynamic interface simulations can be employed to refine the understanding of atomic-scale wetting behavior.

## Figures and Tables

**Table 1:** Experimental measurements and calculated values.

*a* (at. %)	0	10	20	30	40	50	79	99
*θ*_e_ (°)	43 43 [33]	14	12	13	24	31	39	31
*σ*_lv_ (J/m^2^) [29]	1.3	1.0	0.86	0.76	0.69	0.64	0.54	0.49
*σ*_sl_ (J/m^2^)	3.05	3.02	3.16	3.26	3.36	3.46	3.59	3.58
$W_{\mathrm{ad}}^{\mathrm{cal}}$ (J/m^2^)	2.25 2.16 [33]	1.98	1.70	1.50	1.33	1.18	0.95	0.91

**Table 2:** Theoretical calculated values.

*a* (at. %)	0	1.79	3.57	5.36	7.14	23.10	25	45.45	100
*σ*_metal_ (J/m^2^)	1.32	1.22	1.24	1.16	1.13	0.87	0.85	0.76	0.43
*γ*_int_ (J/m^2^)	2.31	2.30	2.86	3.06	3.20	3.58	3.93	3.79	1.83
*W*_ad_ (J/m^2^)	3.05	2.91	2.76	2.67	2.68	1.28	1.09	0.96	2.6

## Data Availability

The original contributions presented in this study are included in the article/Supplementary Materials. Further inquiries can be directed to the corresponding author.

## Supplementary Materials

The following supporting information can be downloaded at: https://www.mdpi.com/article/10.3390/ma18081793/s1, Figure S1: Structures of Cu, Sn, Cr, Cu*_x_*Sn*_y_* (Cu_10_Sn_3_, Cu_3_Sn, Cu_6_Sn_5_), and Cr_3_C_2_: (a) Cu, (b) Sn, (c) Cr, (d) Cu_10_Sn_3_, (e) Cu_3_Sn, (f) Cu_6_Sn_5_, (g) Cr_3_C_2_; Table S1: Space groups and optimized lattice parameters (literature values in parentheses) of the bulk phases. Figure S2: Surface model: (a) Cu(111), (b) Sn(100), (c) Cr(110), (d)Cr_3_C_2_(001), (e) Cu_10_Sn_3_(100)/(010), (f) Cu_10_Sn_3_(001) type 1–3, (g) Cu_3_Sn(100) type 1, 2, (h) Cu_6_Sn_5_(100) type 1–3, (i) Cu_6_Sn_5_(010), (j) Cu_6_Sn_5_(001) type 1–3; Table S2: Surface energy calculation results; Figure S3: The UBER curves of *W*_ad_ with interfacial equilibrium distance; Table S3: *δ*–values of supercell interfaces; Equation (S1): Formula for calculating formation energy (*E*_sub_) of a Cr or Sn atom doped at the Cu||Cr_3_C_2_ interface. References [37,38,43,44,45,46,52,53,54,57,60,61,62] are cited in the Supplementary Materials.
