# Supplementary material for: Effect of Sn Content on Wettability and Interfacial Structure of Cu–Sn–Cr/Graphite Systems: Experimental and First-Principles Investigations
Source: Materials (Basel). 2025 Apr 14;18(8):1793. doi: 10.3390/ma18081793 (PMC12028364; doi:10.3390/ma18081793)
Supplement: Supplementary file 1 [file materials-18-01793-s001.zip › materials-3551763-supplementary.pdf]

Supplementary Materials

1. Bulk model and parameters

Structural models of Cu, Sn, Cr, Cu_xSn_y (ζ–$Cu_{10}Sn_3$, ε–Cu_3Sn, and η'–Cu_6Sn_5) and Cr_3C_2 compounds were obtained from the Materials Project materials database [1] and are shown in Figure S1. ζ–$Cu_{10}Sn_3$, ε–Cu_3Sn, η'–Cu_6Sn_5 are the stabilized structures of $Cu_{10}Sn_3$, Cu_3Sn, and Cu_6Sn_5, respectively [2].

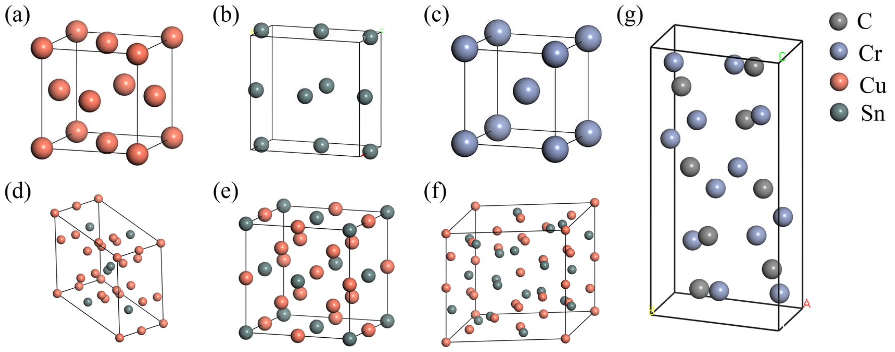

Figure S1. Structures of Cu, Sn, Cr, Cu_xSn_y ($Cu_{10}Sn_3$, Cu_3Sn, Cu_6Sn_5), and Cr_3C_2: (a) Cu, (b) Sn, (c) Cr, (d) $Cu_{10}Sn_3$, (e) Cu_3Sn, (f) Cu_6Sn_5, (g) Cr_3C_2

By comparing the optimized lattice constant values calculated in this work (Table S1) with those from the Refs. [2–5], it is evident that the lattice constants are in good agreement, which indicates that the parameters used in this paper are reasonable and accurate.

Table S1. Space groups and optimized lattice parameters (literature values in parentheses)

Bulk	Space group	Lattice parameters	
		a b c (Å)	α β γ (°)
Cu	$Fm\bar{3}m$	a= 3.63(3.62[3])	α= 90.00
β–Sn	$I4_1/amd$	a= 5.88(5.83)	α= 90.00
		c= 3.20(3.18[4])	γ= 90.00
Cr	$Im\bar{3}m$	a= 2.85(2.88[6])	α= 90.00
$Cu_{10}Sn_3$	$P6_3$	a= 7.38(7.33)	α= 90.00
		c= 7.95(7.86[2])	γ= 120.00
Cu_3Sn	$Fm\bar{3}m$	a= 6.17(6.12[7])	α= 90.00
Cu_6Sn_5	$C12/c1$	a= 11.10(11.02)	α= 90.00
		b= 7.39(7.28)	β= 98.76
		c= 9.97(9.83[5])	γ= 90.00
Cr_3C_2	$Pnma$	a= 5.459(5.53)	α= 90.00
		b= 2.78(2.83)	β= 90.00
		c= 11.41(11.47[8])	γ= 90.00

2. Surface model and parameters

Figure S2 shows the surface model of Cu(111), Sn(100), Cr(110), Cr_3C_2(001), $Cu_{10}Sn_3$(100)/(010), $Cu_{10}Sn_3$(001), Cu_3Sn(100), and Cu_6Sn_5(100)/(010)/(001).

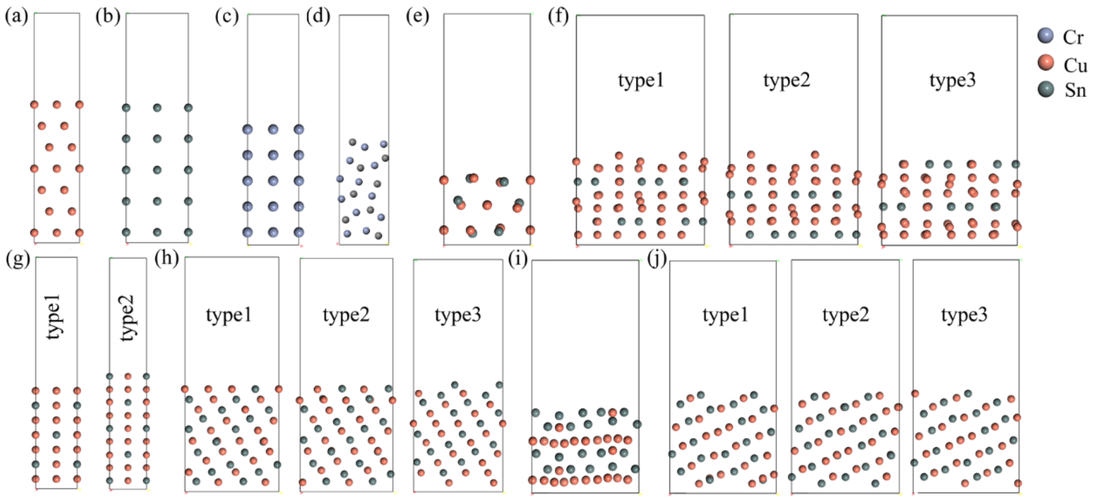

Figure S2. Surface model: (a) Cu(111), (b) Sn(100), (c) Cr(110), (d)Cr_3C_2(001), (e) $Cu_{10}Sn_3$(100)/(010), (f) $Cu_{10}Sn_3$(001) type 1–3, (g) Cu_3Sn(100) type 1, 2, (h) Cu_6Sn_5(100) type 1–3, (i) Cu_6Sn_5(010), (j) Cu_6Sn_5(001) type 1–3

Table S2 lists the surface energies(σ_{metal}) of the surface models shown in Figure S2. Multiple Sn-doped Cu(111) are named $Cu_{56\text{-}k}Sn_k$ (k = 1, 2, 3, 4), where the chosen doping method is simple atomic substitution [9]. A comparison of the σ_{metal} of the different surfaces in Table S2 shows that $Cu_{10}Sn_3$(100), Cu_3Sn(100)type2, and Cu_6Sn_5(001)type2 exhibit lower σ_{metal} values.

Table S2. Surface energy calculation results

Bulk	Surface	Type	σ_{metal} (J/m^2)
Cu	(111)	–	1.32(1.32[10])
Sn	(100)	–	0.43(0.42[11])
Cr	(110)	–	3.18(3.5[6])
Cr_3C_2	(001)		3.99(4.13[8])
$Cu_{55}Sn_1$	–	–	1.22
$Cu_{54}Sn_2$	–		1.24
$Cu_{53}Sn_3$	–		1.16
$Cu_{52}Sn_4$	–		1.13
$Cu_{55}Cr_1$	(111)	–	1.92
$Sn_{49}Cr_1$	(100)	–	0.43
$Cu_{10}Sn_3$	(100)	–	0.87
	(001)	1	1.35
		2	1.18
		3	1.02

Cu_3Sn	(100)	1	1.42
		2	0.85
Cu_6Sn_5	(100)	1	0.88(0.85[12])
		2	0.82
		3	0.98
	(010)	–	0.95(0.93[12])
	(001)	1	0.80
		2	0.76(0.78[12])
		3	0.93

Table S3. ***δ*–values of supercell interfaces**

Interface	Lattice mismatch δ (%)	
	a_1	a_2
$Cu||Cr_3C_2$	6.21	5.71
$Sn||Cr_3C_2$	1.61	5.73
$Cr||Cr_3C_2$	1.62	4.17
$Cu_{10}Sn_3||Cr_3C_2$	6.00	2.84
$Cu_3Sn||Cr_3C_2$	3.74	6.23
$Cu_6Sn_5||Cr_3C_2$	1.60	5.47

3. UBER curves

Figure S3 shows the universal binding energy relation (UBER) curves of interfacial adhesion work W_{ad} with interfacial equilibrium distance (Å). According to Figure S3, it can be seen when the equilibrium distances of the interface are 1.9 Å ($Cu||Cr_3C_2$), 2.2 Å ($Sn||Cr_3C_2$), 1.7 Å ($Cr||Cr_3C_2$), 1.9 Å ($Cu_{10}Sn_3||Cr_3C_2$), and 2.0 Å ($Cu_3Sn(001)||Cr_3C_2$ and $Cu_6Sn_5||Cr_3C_2$), the value of W_{ad} is the largest and the interface stability is the most stable.

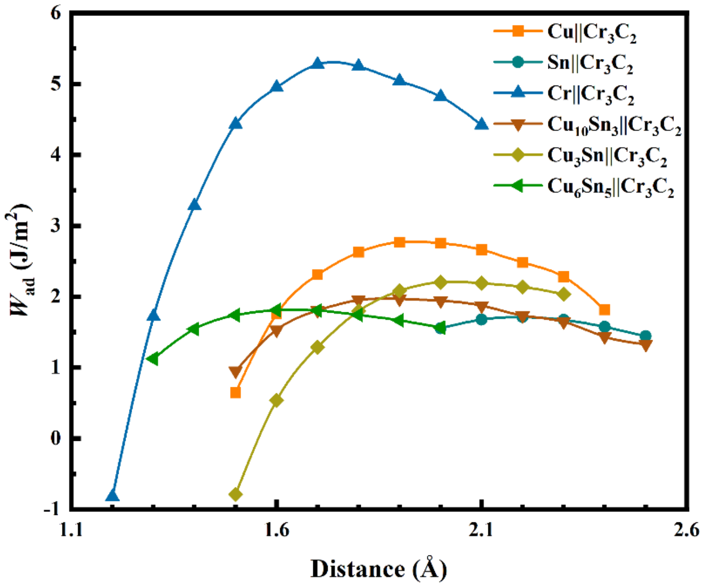

Figure S3. The UBER curves of W_{ad} with interfacial equilibrium distance

4. E_{sub} of a single Cr (Sn) atom doped at the Cu||Cr_3C_2 interface

The formation energy E_{sub} represents the energy required for an atom to occupy the position, and the E_{sub} of a single Cr (Sn) atom doped at different positions within the Cu||Cr_3C_2 interface is used to indicate the ease of doping. The formula for the E_{sub} is shown in Equation (S1) [13]:

$$E_{sub} = E_{\mathrm{total}}^{\mathrm{metal}} + \mu_{\mathrm{X}} - E_{\mathrm{total}}^{\mathrm{Cu}} - \mu_{\mathrm{Cr(Sn)}} \quad \text{(S1)}$$

where E_{sub} is the formation energy, $E_{\mathrm{total}}^{\mathrm{metal}}$ is the total energy of the interface after doping; μ_X is the chemical potential of the substituted atom (here the substituted atoms are Cu atoms in metal or the Cr/C atoms in Cr_3C_2, respectively), $E_{\mathrm{total}}^{\mathrm{Cu}}$ is the total energy of the interface before Cr (Sn) doping and $\mu_{Cr(Sn)}$ is the chemical potential of the Cr(Sn) atom.

5. List of Abbreviations

Abbreviation	Full Term
Cu	Copper
Sn	Tin
Cr	Chromium
C_{gr}	Graphite
C_V	Vitreous carbon

CA	Contact angle
CA_{eq} / θ_e	equilibrium contact angle in references/this study
RPL	Reaction product layer
TL	Triple line
IBP	Interfacial bonding properties
$\sigma_{lv}/\sigma_{metal}$	Experimental/ theoretical calculated value of surface energy of melt
σ_{sl}/γ_{int}	Experimental/ theoretical calculated value of interface energy
W_{ad}^{cal}/W_{ad}	Experimental/theoretical calculated value of adhesion work
PDOS	Partial density of states
DOS	density of states
CDD	Charge density difference
DFT	Density functional theory
CASTEP	Cambridge sequential total energy package
PBE	Perdew-Burke-Ernzerhof
GGA	Generalized gradient approximation
BFGS	Broyden-Fletcher-Goldfarb-Shanno
SEM	Scanning electron microscope
EDS	Energy-dispersive spectroscopy
HRTEM	High-resolution transmission electron microscope
FIB	Focused ion beam
SMEA	Surface Meter Elements Analysis

References

[1] Materials Project: An Open Database of Materials Properties. https://next–gen. materialsproject.org.

[2] A. Leineweber, The Cu–Sn System: A Comprehensive Review of the Crystal Structures of its Stable and Metastable Phases. Journal of Phase Equilibria and Diffusion, (2023) 1–51.

[3] M. Ellner, K. Kolatschek, B. Predel, On the partial atomic volume and the partial molar enthalpy of aluminum in some phases with Cu and Cu_3Au structures, J. Less-Common Met. 170 (1991) 171–184. https://doi.org/10.1016/0022–5088(91)90062–9.

[4] K. N. Tu, Cu/Sn interfacial reactions: thin-film case versus bulk case, Mater. Chem. Phys. 46 (1996) 217–223. https://doi.org/10.1016/S0254–0584(97)80016–8.

[5] A. K. Larsson, L. Stenberg, S. Lidin, The superstructure of domain-twinned η' – Cu_6Sn_5, Acta Cryst B 50 (1994) 636–643. https://doi.org/10.1107/S0108768194004052.

[6] D. I. Bolef, J. de Klerk, Anomalies in the elastic constants and thermal expansion of chromium single crystals, Phys. Rev. 129 (1963) 1063–1067. https://doi.org/10.1103/PhysRev.129.1063.

[7] S. Fürtauer, D. Li, D. Cupid, H. Flandorfer, The Cu–Sn phase diagram, Part I: New experimental results, Intermetallics. 34 (2013) 142–147. https://doi.org/10.1016/j.intermet.2012.10.004.

[8] K. Gu, M. Pang, Y. Zhan, Insight into interfacial structure and bonding nature of diamond(001)/Cr_3C_2(001) interface, J. Alloy. Compd. 770 (2019) 82–89. https://doi.org/10.1016/j.jallcom.2018.08.112.

[9] H. Xie, N. Zhao, C. Shi, C. He, E. Liu, Effects of active elements on adhesion of the Al_2O_3/Fe interface: a first principles calculation, Comput. Mater. Sci. 188 (2021), 110226, https://doi.org/10.1016/j.commatsci.2020.110226.

[10] Z. Wu, M. Pang, Y. Zhan, S. Shu, L. Xiong, Z. Li, The bonding characteristics of the Cu(111)/WC(0001) interface: An insight from first-principle calculations, Vacuum 191 (2021) 110218. https://doi.org/10.1016/j.vacuum.2021.110218.

[11] Z. Ma, H. Li, M. Zhang, Z. Xu, The first principles calculation of the interfacial properties of β–Sn and 4H–SiC, Ordnance. Mater. Sci. Eng. 42 (2019) 17–20. https://doi.org/10.1016/j.commatsci.2020.109608.

[12] Y. Chen, W. Dai, Y. Liu, C. Chen, K.N. Tu, G. Chen, Surface protrusion induced by inter-diffusion on Cu–Sn micro-pillars, Mater. Des. 224 (2022) 111318. https://doi.org/10.1016/j.matdes.2022.111318.

[13] J. Huang, M. Li, Y. Liu, J. Chen, Z. Lai, J. Hu, F. Zhou, J. Zhu, A first-principles study on the doping stability and micromechanical properties of alloying atoms in aluminum matrix, Vacuum 207 (2023) 111596. https://doi.org/10.1016/j.vacuum.2022.111596.
